# Nivolumab-Induced Isolated Neutropenia

**DOI:** 10.7759/cureus.45675

**Published:** 2023-09-21

**Authors:** Aimal Waqas, Javariya Zaffar, Ahmad Jalil, Shayan Butt

**Affiliations:** 1 Internal Medicine, Foundation University Medical College, Islamabad, PAK; 2 Internal Medicine, Rehman Medical College, Peshawar, PAK; 3 Internal Medicine, King Edward Medical University, Lahore, PAK; 4 Internal Medicine, Mayo Clinic, Jacksonville, USA

**Keywords:** isolated neutropenia, severe neutropenia, drug induced neutropenia, immune-checkpoint inhibitor adverse effects, nivolumab related adverse events

## Abstract

Immune checkpoint inhibitors (ICIs) have been discovered to be associated with autoimmune toxicities that may present as dermatologic, gastrointestinal, hepatic, pulmonary, endocrine, and rarely hematologic reactions. Recent studies have also discovered hematological adverse effects as a result of ICI use of which isolated neutropenia is the gravest and the rarest manifestation. Asymptomatic neutropenia cannot be ignored, and close monitoring is, at least, warranted. Severe neutropenia with neutropenic fever needs hospital admission and prompt treatment to avoid further morbidity and/or mortality. In this report, we present a rare case of Grade 4 neutropenia resulting from nivolumab, anti-PD-1 antibody, in a patient diagnosed with invasive bladder cancer. The patient was successfully treated with steroids and supportive measures.

## Introduction

Modulating the immune system, using immune checkpoint inhibitors, instead of targeting cancer cells has yielded a groundbreaking shift in cancer treatment over the recent years [1,2]. Immune checkpoints include but are not limited to PD-1, CTLA-4, LAG3, TIM3, TIGIT, and BTLA. These are inhibitory regulatory proteins that are expressed in normal immune cells and are crucial to maintaining self-tolerance, preventing autoimmunity, and controlling the duration and extent of immune responses in order to minimize tissue damage [3]. These immune checkpoints are often over-expressed on tumor cells or on non-transformed cells within the tumor microenvironment and hinder the ability of the immune system to mount an effective anti-tumor response by negative inhibitory pathway. Checkpoint inhibitors favor anti-tumor properties by simply allowing cellular destruction as they inhibit these checkpoints. At the same time, this results in immune-mediated side effects that are termed immune-related adverse events (irAEs), including pneumonitis, hepatitis, myositis, dermatitis, etc. Hematological irAEs (haem-irAEs) are extremely rare, occurring in up to 1% of cases [4]. Neutropenia, autoimmune hemolytic anemia, and immune thrombocytopenia were the most common types of haem-irAEs during PD-1/PD-L1 treatment in patients registered in three French pharmacovigilance databases [5,6]. In addition to these studies, to the best of our knowledge, there are only a handful of other individual cases of neutropenia, related to ICI treatment published so far [7-14]. In this report, we present a rare case of severe neutropenia resulting from nivolumab, outlining the treatment course done while the patient was hospitalized.

## Case presentation

A 73-year-old male with a past medical history of Parkinson's disease and chronic kidney disease stage II/IIIa was diagnosed with invasive bladder cancer. For this, he underwent radical cystoprostatectomy and was placed on the checkpoint inhibitor nivolumab. He received nivolumab once a month and tolerated the first four doses without any major side effects. Eventually, he presented with intermittent hematuria and fever to the oncology clinic 10 days after his fifth dose of nivolumab. Lab work was significant for an absolute neutrophil count (ANC) of 0.02 x 10^9^/L. He was subsequently admitted to the hospital for evaluation and treatment of neutropenic fever.

The patient was on carbidopa-levodopa, duloxetine, and simvastatin at home. On physical examination, vitals showed a heart rate of 75 beats per minute, a respiratory rate of 18 breaths per minute, a temperature of 37.4 °C, and a blood pressure of 120/70 mmHg. He did not appear to be in any distress and appeared at the stated age. Small punctate white lesions, superimposed on erythematous mucosa, were seen on the hard palate. Mild bradykinesia was present, and a urostomy with the attached bag was intact in the right lower quadrant. The abdomen was soft, non-tender, and non-distended.

On admission, his lab results showed his Hgb at 11.2 g/dL, his WBC at 1.7 x 10^9^/L, and his ANC at 0.02 x 10^9^/L. The basic metabolic panel and hepatic function panel did not show any abnormal values. A CT scan of the abdomen and pelvis without contrast did not indicate nephrolithiasis or hydronephrosis.

He was empirically started on cefepime, and blood and urine cultures were sent. Tests for B12, folic acid, and copper levels were also conducted, along with serologies for hepatitis A, B, and C, human immunodeficiency virus (HIV), herpes simplex virus (HSV), cytomegalovirus (CMV), and Epstein-Barr virus (EBV), and parvovirus B19, as potential causes of neutropenia and fever. A swab for HSV PCR was obtained from the hard palate. Filgrastim 480 mcg was started with a goal ANC of 1.0 x 10^9^/L, and the hematology service was consulted. All serologies returned negative for active pathogens, and there was no growth on cultures. Consequently, cefepime was discontinued.

**Figure 1:**
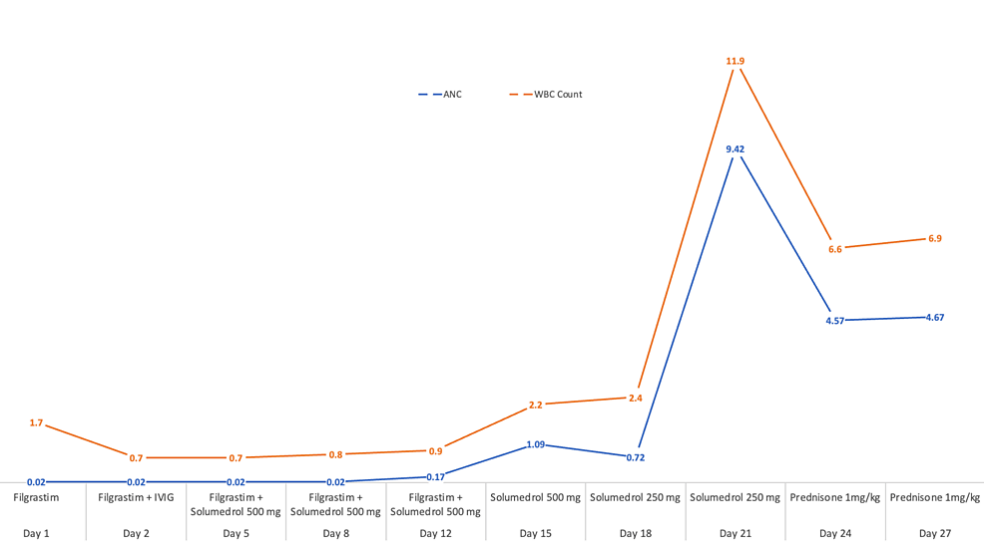
Therapeutic intervention and response x-axis: pharmacological intervention and day number; y-axis: ANC and WBC x 10^9^/L; Red: total WBC count; Blue: absolute neutrophilic count (ANC)

The patient showed no improvement in the ANC even four days after starting filgrastim. He was initiated on IVIG at a dosage of 400 mg/kg and completed a four-day course, but his neutropenia persisted. Given that both IVIG and filgrastim failed to induce any improvement, he was subsequently started on methylprednisolone 500 mg daily. His ANC gradually improved, reaching 1.09 x 10^9^/L on the 15th day of admission, which was also the 9th day of steroid therapy (Table 1). After maintaining an ANC above 1 x 10^9^/L for two additional days, filgrastim was discontinued and the dose of methylprednisolone was reduced to 250 mg daily. With persistent stabilization of both WBCs and ANC, he was then started on 120 mg (1 mg/kg) of oral prednisone daily. Notably, there was a brief drop in ANC for two days when transitioning from 500 mg to 250 mg of methylprednisolone, but no dose adjustments were made and a sustained increase in ANC was achieved (Figure 1).

**Table 1:** Lab Flowsheet

	WBC Count (3.4-9.6 × 10^9^/L)	Lymphocytes (0.95-3.07 × 10^9^/L)	Platelets (135-317 × 10^9^/L)	Neutrophils (1.56-6.45 × 10^9^/L)	Hemoglobin (13.2–16.6 g/dL)
Day 1	1.7	1.06	155	0.02	11.2
Day 4	1.0	0.87	143	0.02	10.5
Day 8	0.8	0.56	236	0.02	10.5
Day 15	2.2	0.84	165	1.09	11.9
Day 18	2.4	0.95	154	0.72	11.9
Day 21	11.9	1.39	61	9.42	13.2
Day 24	6.6	1.46	110	4.57	13.0

A persistent ANC count of more than 1000/mm^3 ^or 1.0 x 10^9^/L was achieved on the 21st day of admission and the patient was discharged to a rehab facility on a prednisone taper with sulfamethoxazole-trimethoprim for PCP prophylaxis. 

## Discussion

One of the major mechanisms of evasion of cell death by tumor cells is by utilization of immune checkpoint proteins present on the surface of T-cells. Tumor cells have partner proteins on their surface that bind to checkpoint proteins on T-cells to send an "off" signal to prevent the immune system from destroying cancer cells. In the pharmacological arsenal, there are ICIs that work by blocking these checkpoint proteins from binding with their partner proteins. Thus, the "off" signal is never generated, which in turn allows T-cells to kill tumor cells.

The most studied immune checkpoints are Programmed Cell Death-1 (PD-1)/Programmed Cell Death Ligand-1 and Cytotoxic T-Lymphocyte Antigen 4 (CTLA-4). Nivolumab and pembrolizumab inhibit PD-1 and ipilimumab inhibits CTLA-4. Any and all of these ICI inhibitors may carry adverse effects. Heme irAEs have been more commonly associated with nivolumab- and pembrolizumab-based therapies (35% and 33%, respectively) than with ipilimumab (16%) or ipilimumab with nivolumab (13%) [15]. Based on our literature review, nivolumab stands out to be the most common contributor to ICI-induced neutropenia, but the exact biochemical cause for this might be unknown [6,16].

According to the National Cancer Institute Common Terminology Criteria for Adverse Events, chemotherapy-induced neutropenia (CIN) can be divided into four grades: Grade 1 with an ANC of 1500-2000 cells/mm^3^; Grade 2 with an ANC of 1000-1500 cells/mm^3^; Grade 3 with an ANC of 500-1000 cells/mm^3^; and Grade 4 with an ANC <500 cells/mm^3^. The median onset of ICI-mediated neutropenia is 10 to 11 weeks, with a median duration (at grade 2 or worse) of 13 to 16.5 days [1]. In our case, the patient was reported to have Grade 4 neutropenia 20 weeks later with a median duration of 21 days. A recent analysis called Vigibase utilized data from WHO’s pharmacovigilance database and focused on 168 cases of haem-irAEs. It revealed that 67% of these cases exhibited neutropenia along with fever, similar to the symptoms observed in our patient [5]. The report also highlighted that 23% of the cases experienced non-heme irAEs. However, our patient exhibited neutropenia as a sole manifestation without any other associated immune-mediated adverse effect.

It is always imperative to rule out any viral agent as a cause of neutropenia or any cytopenia. Negative viral studies and improvement in neutrophil count with steroids highlighted ICI use as being the only offender for the presentation. Despite certain contradictory information surrounding the use of systemic corticosteroids and their potential to elevate the risk of infection, it is now widely acknowledged that patients experiencing irAEs should be started on steroid therapy which is primarily based on collective knowledge gained through large, randomized trials and clinical experience since no prospective study has been conducted to determine the best approach for managing these toxicities [6, 8]. 

In our case of Grade 4 neutropenia caused by nivolumab, corticosteroid therapy was found to be very successful. Steroids are thus far the mainstay of treatment and have been reported by Baleiras et al. [2], Akhtari et al. [6], Tabchi et al. [8], and Meti et al. [12]. It was interesting to note that IVIG and GM-CSF (Filgrastim) failed to produce any response, which is consistent with Ban-Hoefen et al. [7], Barbacki et al. [10], and Bryant et al. [17]. In some of these cases where steroids, IVIG, and GM-CSF failed to produce any desirable effect, the use of immunosuppressants such as cyclosporine [7] and tocilizumab [17] is reported. In very rare instances as reported by Bryant et al. [17], patients had to undergo stem cell transplantation for treatment of refractory neutropenia and complications from neutropenia.

## Conclusions

Though the use of ICIs that specifically target the immune regulatory checkpoint receptors such as PD-1 (through pembrolizumab, nivolumab, cemiplimab, and dostarlimab), PD-L1 (through atezolizumab, avelumab, and durvalumab), and CTLA-4 (through ipilimumab) has revolutionized the cancer treatment, it has also caused a significant decline in health and premature cessation of treatment due to irAEs occurrence. Isolated neutropenia secondary to ICIs poses a potential threat to patients’ lives, particularly given the limited amount of literature available on the subject. Recognition of this uncommon and severe side effect underlines the significance of its timely detection and immediate treatment with high-dose steroids.

## References

[1] Petrelli F, Ardito R, Borgonovo K, Lonati V, Cabiddu M, Ghilardi M, Barni S (2018). Haematological toxicities with immunotherapy in patients with cancer: a systematic review and meta-analysis. Eur J Cancer.

[2] Miranda Baleiras M, Vasques C, Pinto M, Miranda H, Martins A (2022). Pembrolizumab-induced autoimmune grade 4 neutropenia in a patient with advanced bladder cancer: a case report. Cureus.

[3] Ghanem P, Marrone K, Shanbhag S, Brahmer JR, Naik RP (2022). Current challenges of hematologic complications due to immune checkpoint blockade: a comprehensive review. Ann Hematol.

[4] Davis EJ, Salem JE, Young A (2019). Hematologic complications of immune checkpoint inhibitors. Oncologist.

[5] Delanoy N, Michot JM, Comont T (2019). Haematological immune-related adverse events induced by anti-PD-1 or anti-PD-L1 immunotherapy: a descriptive observational study. Lancet Haematol.

[6] Akhtari M, Waller EK, Jaye DL, Lawson DH, Ibrahim R, Papadopoulos NE, Arellano ML (2009). Neutropenia in a patient treated with ipilimumab (anti-CTLA-4 antibody). J Immunother.

[7] Ban-Hoefen M, Burack R, Sievert L, Sahasrabudhe D (2016). Ipilimumab-induced neutropenia in melanoma. J Investig Med High Impact Case Rep.

[8] Tabchi S, Weng X, Blais N (2016). Severe agranulocytosis in a patient with metastatic non-small-cell lung cancer treated with nivolumab. Lung Cancer.

[9] Woźniak S, Mackiewicz-Wysocka M, Krokowicz Ł, Kwinta Ł, Mackiewicz J (2015). Febrile neutropenia in a metastatic melanoma patient treated with ipilimumab - case report. Oncol Res Treat.

[10] Barbacki A, Maliha PG, Hudson M, Small D (2018). A case of severe pembrolizumab-induced neutropenia. Anticancer Drugs.

[11] Sun Y, Lee SK, Oo TH, Rojas-Hernandez CM (2018). Management of Immune-mediated cytopenias in the Era of Cancer Immunotherapy: a report of 4 cases. J Immunother.

[12] Meti N, Petrogiannis-Haliotis T, Esfahani K (2018). Refractory neutropenia secondary to dual immune checkpoint inhibitors that required second-line immunosuppression. J Oncol Pract.

[13] Turgeman I, Wollner M, Hassoun G, Bonstein L, Bar-Sela G (2017). Severe complicated neutropenia in two patients with metastatic non-small-cell lung cancer treated with nivolumab. Anticancer Drugs.

[14] Brahmer JR, Tykodi SS, Chow LQ (2012). Safety and activity of anti-PD-L1 antibody in patients with advanced cancer. N Engl J Med.

[15] Naqash AR, Appah E, Yang LV (2019). Isolated neutropenia as a rare but serious adverse event secondary to immune checkpoint inhibition. J Immunother Cancer.

[16] Kanai O, Nakatani K, Fujita K, Okamura M, Mio T (2021). No need to hesitate: immune-related neutropenia and thrombocytopenia that improved by corticosteroids. Respirol Case Rep.

[17] Bryant AR, Perales MA, Tamari R, Peled JU, Giralt S (2018). Severe pembrolizumab-associated neutropenia after CD34(+) selected allogeneic hematopoietic-cell transplantation for multiple myeloma. Bone Marrow Transplant.

